# High Frequency of Copy Number Variations and Sequence Variants at *CYP21A2* Locus: Implication for the Genetic Diagnosis of 21-Hydroxylase Deficiency

**DOI:** 10.1371/journal.pone.0002138

**Published:** 2008-05-14

**Authors:** Silvia Parajes, Celsa Quinteiro, Fernando Domínguez, Lourdes Loidi

**Affiliations:** 1 Fundación Pública Galega de Medicina Xenómica (Unidad de Medicina Molecular), Hospital Clínico Universitario, Santiago de Compostela, A Coruña, Spain; 2 Departamento de Fisiología, Universidad de Santiago de Compostela, Santiago de Compostela, A Coruña, Spain; Stanford University, United States of America

## Abstract

**Background:**

The systematic study of the human genome indicates that the inter-individual variability is greater than expected and it is not only related to sequence polymorphisms but also to gene copy number variants (CNVs). Congenital Adrenal Hyperplasia due to 21-hydroxylase deficiency (21OHD) is the most common autosomal recessive disorder with a carrier frequency of 1∶25 to 1∶10. The gene that encodes 21-hydroxylase enzyme, *CYP21A2,* is considered to be one of the most polymorphic human genes. Copy number variations, such as deletions, which are severe mutations common in 21OHD patients, or gene duplications, which have been reported as rare events, have also been described. The correct characterization of 21OHD alleles is important for disease carrier detection and genetic counselling

**Methodology and Findings:**

*CYP21A2* genotyping by sequencing has been performed in a random sample of the Spanish population, where 144 individuals recruited from university students and employees of the hospital were studied. The frequency of *CYP21A2* mutated alleles in our sample was 15.3% (77.3% were mild mutations, 9% were severe mutations and 13.6% were novel variants). Gene dosage assessment was also performed when *CYP21A2* gene duplication was suspected. This analysis showed that 7% of individuals bore a chromosome with a duplicated *CYP21A2* gene, where one of the copies was mutated.

**Conclusions:**

As far as we know, the present study has shown the highest frequency of 21OHD carriers reported by a genotyping analysis. In addition, a high frequency of alleles with *CYP21A2* duplications, which could be misinterpreted as 21OHD alleles, was found. Moreover, a high frequency of novel genetic variations with an unknown effect on 21-hydroxylase activity was also found. The high frequency of gene duplications, as well as novel variations, should be considered since they have an important involvement in carrier testing and genetic counseling.

## Introduction

Congenital Adrenal Hyperplasia due to 21-hydroxylase deficiency (21OHD) (OMIM +201910) is the most common autosomal recessive disorder. This enzymatic deficiency impairs synthesis of cortisol and/or aldosterone from cholesterol in the adrenal cortex. The severe deficiency of steroid 21-hydroxylase enzyme (21OH; EC 1.14.99.10) results in the classic form of the disease, which is divided firstly, into the salt-wasting form, where neither cortisol nor aldosterone is synthesized; and secondly, into the simple virilizing form, where a residual activity of 21OH allows aldosterone synthesis. The moderate deficiency of 21OH results in the non classic form of the disease (NC21OHD), which is characterized by precocious pseudopuberty, acne, hirsutism and oligomenorrhea, although phenotypical expression is highly variable [1]–[3].

The incidence of the classic form of the disease is 1∶23000–1∶10000 depending on the populations studied [4]–[8] and the incidence of the NC21OHD is 1∶500–1∶100 in most populations. It can be more frequent in other populations like in the Ashkenazi Jews with a 1∶27 incidence [2], [9]. The carrier frequency of 21OHD, estimated on the basis of newborn screening, is between 0.011 and 0.020 for severe mutations and 0.08 to 0.18 for mild ones. On the other hand, genotyping analyses done in New Zealanders and Middle Europeans have shown higher frequencies for carriers of severe mutations (0.040–0.055) and lower for mild ones (0.02–0.04) [4], [10]. Most 21OHD patients are carriers of deletions of the gene encoding 21OH, *CYP21A2,* or of any of the 9 most frequent point mutations derived from the non-functional *CYP21A1P* pseudogene. The rest are due to rare genetic variants, descriptions of which are continuously increasing [11]–[20].

**Figure 1:**
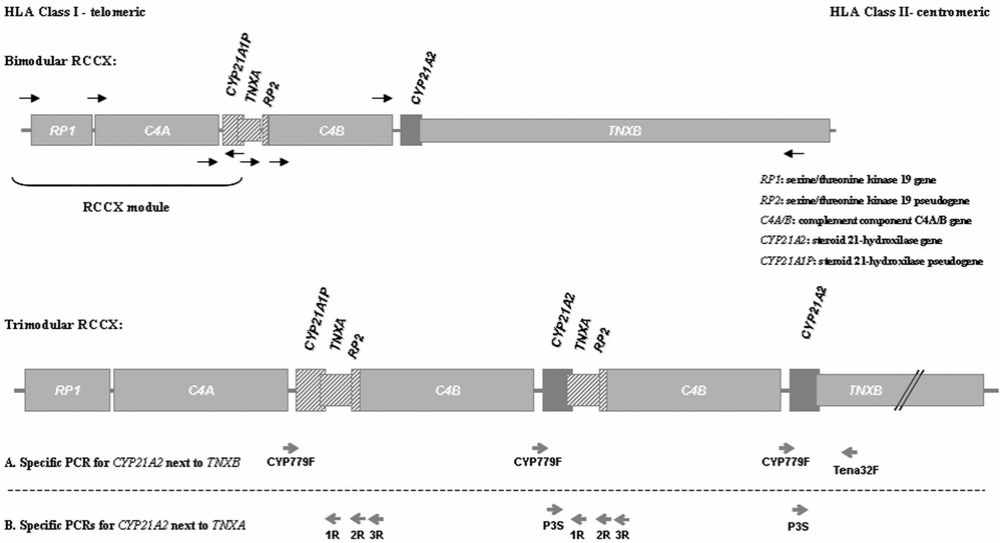
Organization of RCCX module and Copy Number Variations at chromosome 6p21. Black horizontal arrows denote gene orientation. A. Specific primers used for the amplification of *CYP21A2* next to *TNXB.* B. Specific primers used for the amplification of *CYP21A2* next to *TNXA*. Primer binding sites for each primer are indicated by grey horizontal arrows.

The *CYP21A2* gene is 98% homologous to the *CYP21A1P* pseudogene in its coding sequence and 96% in introns. Both lie in the Major Histocompatibility Complex at chromosome 6p21.3, which is a complex organization of genes with variation in gene copy number and size [1], [21]. They constitute a genetic unit together with neighboring genes *RP1, C4, TNXB,* and their truncated pseudogenes *RP2* and *TNXA.* This genetic unit is termed RCCX module (*RP-C4-CYP21-TNX*) and constitutes a highly variable stretch of DNA of approximately 30 kb [22] (Figure 1). Most chromosomes bear two of these modules, one with the *CYP21A1P* pseudogene and the other with the *CYP21A2* gene. Monomodular and trimodular haplotypes have also been described [22]–[27].

The high genetic variability at *CYP21A2* locus makes the characterization of 21OHD alleles difficult, and, hence, complicates disease carrier detection and genetic counseling. In this paper, an in-depth study of the *CYP21A2* gene in a group of 144 individuals is described. We found an unexpectedly high 21OHD carrier frequency, *CYP21A2* gene duplications and *CYP21A2* novel genetic variations. The results of the current work should be considered for 21OHD genetic diagnosis and counseling.

## Results

### Copy Number Variations: frequency of *CYP21A2* gene duplications

The *CYP21A2* gene dosage assessment by Real-Time PCR and by the specific amplification of each duplicated gene showed that 8 out of the 144 individuals were carriers of *CYP21A2* gene duplication with one of the copies p.Gln318X mutated (UniProtKB ID P08686). In all the cases, the mutation was in linkage disequilibrium with two uncommon genetic variants, a G>A change in intron 2 at position -79 and a C>T change at nucleotide 13 in the 3′ UTR region (c.293-79G>A and c.*13C>T, respectively, GenBank Ref. ID NM000500.5). The PCRs for the amplification of the *CYP21A2* duplicated genes confirmed the duplication. The specific sequencing of each gene showed the severe mutation on the *CYP21A2* next to the *TNXB* gene, and the presence of a wild-type *CYP21A2* next to the *TNXA* pseudogene.

Two other gene duplications were found by Real-Time PCR in two chromosomes bearing a chimeric *CYP21A1P/CYP21A2*. The duplications had been suspected by the presence of heterozygosity in the PCR products of a *CYP21A2* fragment despite the fact that both individuals were carriers of p.Ile236Asn, p.Val237Glu, and p.Met239Lys mutations, which prevented the specific primer from its binding. The PCRs for the specific amplification of each *CYP21A2* duplicated gene confirmed the duplication. The sequencing of each gene showed the chimeric *CYP21A1P/CYP21A2* gene next to the *TNXA* pseudogene, and the presence of a wild-type *CYP21A2* next to the *TNXB* gene.

The total number of duplications found in our sample results in a duplication allele frequency of 0.035, which meant a 7% frequency of carriers. Most of them bore the p.Gln318X mutation in one of the *CYP21A2* genes.

### Sequence Variations: frequency of putative disease-causing alleles

A total of 22 *CYP21A2* mutated alleles were found (Table 1), which indicated that 16% of individuals were mutation carriers. No significant differences were found between males and females. Seventeen of these *CYP21A2* genetic variants were known to maintain a residual 21-hydroxylase enzyme (21OH) activity, 2 completely impaired it, and 3 were novel variants with an unknown effect on the 21OH activity (Table 1).

**Table 1:** *CYP21A2 g*enetic variations found in a random sample of 144 Spaniards.

Type of Variation!!	Genetic variation†	Sequence Variation at protein level£	21OH& activity#	N° of alleles (allele frequency)
Mild	c.844G>T	p.Val281Leu	20–50%	16 (0.0555)
	c.91C>T	p.Pro30Leu	30–60%	1 (0.0035)
Severe	Conversion	*	0%	1 (0.0035)
	c.874G>A	p.Gly291Ser	0.8%	1 (0.0035)
Novel	c.1996C>A	p.Thr443Asn	unknown	1 (0.0035)
	c.553G>A	p.Asp184Asn	unknown	1 (0.0035)
	c.69G>T	p.Trp22Cys	unknown	1 (0.0035)
Gene duplications	c.[Conversion;Wt]	p.[§;Wt]	100%	1 (0.0035)
	c.[Conversion;Wt]	p.[‡;Wt]	100%	1 (0.0035)
	c.[955C>T;Wt]	p.[Gln318X;Wt]	100%	8 (0.0278)

!! Single Nucleotide Polymorphisms and the insertion of CTG encoding Leu10 are not reported in this table.

† GenBank Ref. ID NM_000500.5.

£ UniProtKB ID P08686.

& 21OH: 21-hydroxylase.

# Data obtained from White et al. 2000.

Conversion: Chimeric *CYP21A1P/CYP21A2*.

* p.[Pro30Leu;Gly110del8nt].

§ p.[Ile172Asn;Asp183Glu;Ile236Asn;Val237Glu;Met239Lys;Val281Leu;Phe306ins1T].

‡ p.[Asp183Glu;Ile236Asn;Val237Glu;Met239Lys;Val281Leu;Phe306ins1T;Arg356Trp].

#### Mild mutations

The allele frequency for mild mutations was 0.059. The most frequent was p.Val281Leu, which was carried by 11% individuals.

#### Severe mutations

The allele frequency for severe mutations was 0.007. A chimeric *CYP21A1P/CYP21A2* gene was carried by an individual and p.Gly291Ser was carried by another.

#### Novel genetic variations

**Figure 2:**
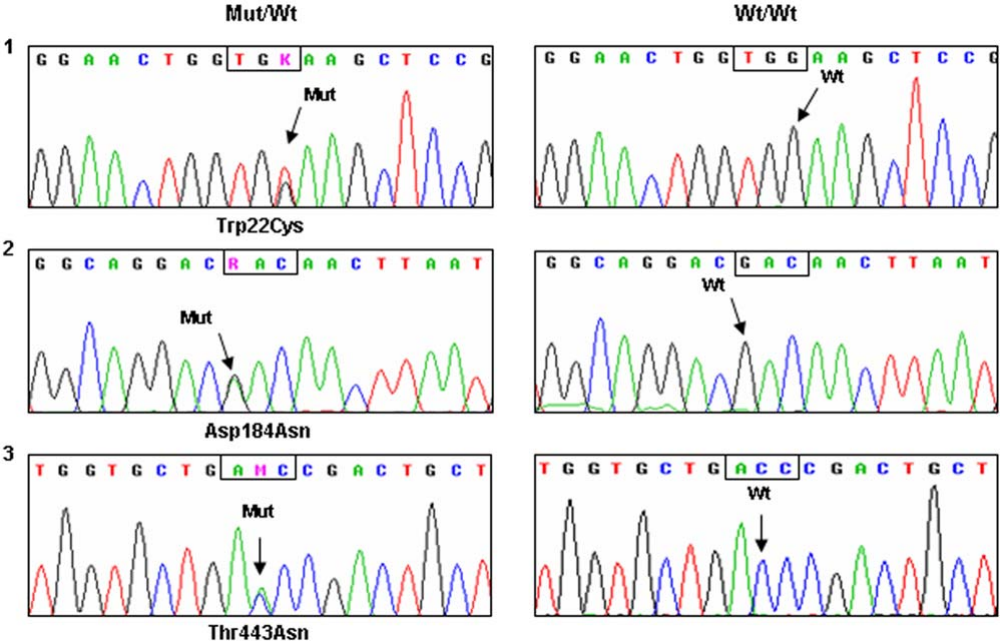
Electropherograms obtained for the three novel mutations in *CYP21A2* gene and the corresponding wild-type alleles. 1. The base change from G to T at position 69 leads to the substitution of tryptophan 22 by cysteine; 2. The base change from G to A at position 553 replaces aspartic acid 184 by asparagine; 3. The base change from C to A at positition 1996 leads to the substitution of threonine 443 by asparagine. (GenBank Ref. ID NM_000500.5; UniProtKB ID P08686). Wt: wild-type; Mut: mutated.

In our sample, 0.010 alleles carried novel genetic variants: p.Trp22Cys, p.Asp184Asn, and p.Thr443Asn. Each of these was found in different individuals (Figure 2). The effect of these mutations on the 21OH activity is unknown.

### *CYP21A2* gene polymorphisms and haplotypes

In the 288 chromosomes analyzed, a total of 79 different genetic variants were found. They were distributed in the vicinity 5′ and 3′ UTR of the *CYP21A2* gene as well as in introns and exons. Haplotype inference from data obtained for the 20 most polymorphic genetic variations enabled the identification of 75 different haplotypes. Most of them appeared only once, but others were recurrent. These latter were the haplotype associated to the p.Gln318X mutation, which is unique, or the haplotype associated to the p.Val281Leu mutation, which was present in 75% of these mutated alleles (data not shown).

## Discussion

The estimated frequency of 21OHD allele carriers among different populations is 1.8% when inferred from the hormonal neonatal screening data [1], [28], which is designed to detect mostly the classic form of 21OHD. The frequency determined by *CYP21A2* genotyping, which also detects NC21OHD, is from 4% to 10% [4], [10]. It has been reported that 14% of classic forms and 84% of non-classic forms are missed on the neonatal screening performed during the first days of life [29]. There are no 21OHD carrier frequency data for the Spanish population based on *CYP21A2* genotyping. In this study, the complete analysis of the *CYP21A2* gene has been performed in 144 random volunteers. The object of the study was firstly, to determine the frequency of 21OHD carriers and secondly, to characterize rare RCCX modules in alleles with duplicated *CYP21A2.* Although the sample size was small, our data concord with previously published studies (8); it may have been preferable to have determined the carrier frequency using a larger sample; this, however, was both time-consuming and expensive.

Copy number variations (CNVs) have been described for the RCCX locus: the presence of two RCCX modules is the standard and the presence of one, three or even four modules are rare arrangements. The trimodular organization accounts for only 14% of the chromosomes in population studies [23] and the majority carry two copies of the *CYP21A1P* pseudogene and one copy of the *CYP21A2* gene. The trimodular haplotype has also been described with two copies of the *CYP21A2* gene and one copy of the *CYP21A1P* pseudogene. This latter haplotype has been described, in some carriers of the p.Gln318X mutation and chimeric *CYP21A1P/CYP21A2* genes, as a rare event [23]–[27]. Our series, however, have shown a high frequency of this arrangement, 7% of individuals. The specific amplification and sequencing of each duplicated gene localized the p.Gln318X mutation at the *CYP21A2* gene next to *TNXB* gene and it showed a wild-type *CYP21A2* at the 3′UTR of *TNXA* pseudogene. Thus, PCR approaches [30] based on the specific amplification of *CYP21A2* like genes next to *TNXB* would fail in the detection of the wild-type gene and would misdiagnose these duplicated alleles as pathologic, which they are certainly not.

The duplicated *CYP21A2* alleles found bear one inactivated gene due to the severe mutations and a wild-type gene which abolishes the pathological effect of the mutation. Furthermore, *CYP21A2* duplications have recently been reported as a risk factor for *de novo* mutations in the offspring [13]. Hence, an awareness of the presence of these types of gene duplications is important if a misdiagnosis is to be avoided. A possible misdiagnosis could be the erroneous identification of a wild type allele as pathological. This is critical for the molecular diagnosis of 21OHD, as well as for prenatal testing and for genetic counseling.

We found that 15.3% of individuals from our sample were carriers of *CYP21A2* mutations including novel variants. As far as we know, this is the highest carrier frequency described so far.

The percentage of individuals who were carriers of NC21OHD was 12%. The application of Hardy-Weinberg equilibrium to our frequency data, estimated the incidence of the mild form of the disease in 1∶225. This value is a more precise estimation than the incidence estimated by clinical diagnosis because the latter may underdiagnose patients with mild phenotypes [2]. The frequency of NC21OHD carrier found by genotyping in unselected Middle Europeans and New Zealanders was lower than ours [4], [10]. This may reflect ethnic differences between the populations studied. Although it should be noted that we sequenced the complete *CYP21A2* gene whereas the other genotyping based studies only analyzed the 9–11 most common mutations.

In this investigation, as the Middle Europeans and New Zealanders studies, the p.Val281Leu mutation was the most common mutation among NC21OHD alleles (94% in our sample). This was consistent with the reported mutational spectrum of non-classic patients [2], [18].

The frequency of carriers of severe mutations found (1.4%) corresponded with the incidence of the classic form observed in ours, as well as other populations. It is also similar to data obtained from genotyping in New Zealanders [10], [21]. Nevertheless, it is much lower than the frequency described for Middle European population (5.5%) [4]. This may reflect a different incidence of classic 21OHD when European populations were compared [6]–[8]. The frequency of *CYP21A2* deletions and chimeric *CYP21A1P/CYP21A2* genes found was lower than the 15–20% described by other authors [31], [32]. However, it corresponded with our previous work [18] and it is similar to the frequency observed in classic 21OHD patients from Italy, France or England [33]–[35].

Three novel genetic variants were found, (p.Trp22Cys, p.Asp184Asn, and p.Thr443Asn) which account for approximately 1% of chromosomes. Two of them, p.Trp22Cys and p.Thr443Asn, are predicted to damage 21-hydroxylase activity (PolyPhen tool (http://genetics.bwh.harvard.edu/pph/)). The high frequency of novel genetic variants shows the importance of sequencing the whole *CYP21A2* gene before it can be considered as wild-type. In some cases, the effect of novel genetic variants may be deduced from the phenotype of the patients. Nevertheless, *in vitro* functional studies are needed to ascertain them and, if possible, they should be made before considering a variation as a pathological mutation or as a functional polymorphism.

The *CYP21A2* analysis in our Spanish random population has confirmed that this gene is one of the most polymorphic human ones, as it has already been described by Cargill and co-workers [36]. We found up to 79 different genetic variations at this locus. Twenty of these genetic variations showed a minor allele frequency higher than 0.01. These polymorphic variations rendered up to 73 different haplotypes (data not shown), most of them occurred only once.

In conclusion, as far as we know, the present study has shown the highest frequency of 21OHD carriers reported by a genotyping analysis. In addition, *CYP21A2* gene duplications with one of the copies mutated have been found to be a common event. Moreover, a high frequency of novel genetic variants with an unknown effect on 21OH activity was found and represents a source of uncertainty in 21OHD genetic diagnosis. The number of novel variants, as well as gene duplications found here, could be as high in other populations and should be considered when the 21OHD genetic diagnosis and genetic counseling is carried out.

## Materials and Methods

### DNA samples

The *CYP21A2* gene was studied in a random sample of 144 individuals (95 females, 49 males) recruited from the university students and employees of the hospital. Information about their parents' and grandparents' geographic origins was provided. All these individuals gave informed consent for the analysis, and the procedures were conducted according to the principles expressed in the Declaration of Helsinki.

### Genetic analysis

DNA was extracted from peripheral blood leucocytes by standard procedures.

#### Sequencing

The *CYP21A2* gene and the closest 5′ and 3′ UTR were sequenced after being amplified in two overlapping fragments by the polymerase chain reaction method as previously described [18].

#### CYP21A2 copy number assessment:

For the *CYP21A2* gene dosage assessment, 100 ng of DNA were used. The analytical procedure was based on a Real-Time PCR method, using specific primers for the amplification of the *CYP21A2* gene, and TaqMan® (Applied Biosystems) probes as previously described [37].

#### Specific amplification of each duplicated CYP21A2 gene

Different DNAs previously characterized by southern blot and the Real-Time PCR method were used as controls for the following PCRs

A.- For the amplification of the *CYP21A2* at the 3′ UTR of *TNXB*, the strategy described by Lee HH and co-workers was followed [30]. This is based on the amplification of an 8515 bp fragment by the use of a reverse primer (Tena32F), which anneals specifically to exon 32 of *TNXB*. The forward primer (CYP779F) is common for the 5′ UTR of *CYP21A2* and *CYP21A1P*. The samples with a single copy of *CYP21A2* on a chromosome, as well as those samples with two copies of *CYP21A2* on the same chromosome, have to be positive for this PCR.

B.- For the amplification of the *CYP21A2* gene at the 3′ UTR of *TNXA*, we designed three different PCRs using a forward primer (P3S) that anneals specifically to exon 3 of *CYP21A2* and: B1, a reverse primer (1R), which anneals to *TNXA* producing a fragment of 4611 bp. B2, with reverse primer (2R), which specifically anneals to *RP2* and forward primer (P3S) resulting in a 7042 bp fragment and B3, with a reverse primer (3R), which anneals to *C4* producing a 7475 bp fragment. (Figure 1) The samples with a single copy *CYP21A2* on a chromosome have to be negative for these PCRs, while samples with two *CYP21A2* copies on a chromosome have to be positive for these PCRs. (Table S1. PCR conditions under request).

The duplicated *CYP21A2* alleles found were subjected to these PCRs and the products obtained were sequenced in order to know whether the p.Gln318X mutated copy and the chimeric *CYP21A1P/CYP21A2* genes were next to *TNXB* or to *TNXA.*

#### Haplotype construction

The Bayesian statistical method implemented in the program PHASE v2.1.1 was used for the haplotype construction. Only DNA variations, pathologic or not, with a minor allele frequency higher than 0.01 were considered. This selection criterion resulted in 20 genetic variations distributed all over the *CYP21A2* gene, as well as the closest 5′ and 3′ UTR. Eighteen of these variations were biallelic markers, and two were triallelic markers: rs6451 and rs6467. It was also included the polymorphic insertion of an extra leucine in exon 1, and the deletion of a G at position -106 of intron 2 (rs41315224).

## Supporting Information

Table S1 Sequences of primers.
(0.03 MB DOC)
