# Supplementary material for: High Frequency of Copy Number Variations and Sequence Variants at CYP21A2 Locus: Implication for the Genetic Diagnosis of 21-Hydroxylase Deficiency
Source: PLoS One. 2008 May 14;3(5):e2138. doi: 10.1371/journal.pone.0002138 (PMC2364643; doi:10.1371/journal.pone.0002138)
Supplement: Table S1 — Sequences of primers. (0.03 MB DOC) [file pone.0002138.s001.doc]

Sequences of primers used for the specific amplification of each *CYP21A2* duplicated gene.

| **Primer** | **Sequence** |
| --- | --- |
| **CYP779F** | 5’-AGG TGG GCT GTT TTC CTT TCA-3’ |
| **Tena32F** | 5’-CTG TGC CTG GCT ATA GCA AGC C-3’ |
| **P3S** | 5’-TTG TCC TTG GGA GAC TAC TCC-3’ |
| **Primer 1R** | 5’-CCA GAA ACT CCA GGT GGG AGT-3’ |
| **Primer 2R** | 5’-CTT TGC CTT CCG GAC CAT GCC-3’ |
| **Primer 3R** | 5’-GCT CAA GCT GTG AGG AGA ACT-3’ |
